# Advancing Our Understanding of Surface Water Temperature Dynamics in Transitional Environments through in Situ, Satellite, and Hydrodynamic Modeling

**DOI:** 10.1021/acsestwater.5c00583

**Published:** 2025-11-26

**Authors:** Nagendra Jaiganesh Sankara Narayanan, Debora Bellafiore, Francesca De Pascalis, Michol Ghezzo, Claire Miller, Marian Scott, Federica Braga, Evangelos Spyrakos, Andrew Tyler

**Affiliations:** † Earth and Planetary Observation Sciences (EPOS), Biological and Environmental Sciences, Faculty of Natural Sciences, 7622 University of Stirling, Stirling, United Kingdom FK9 4LA; ‡ Institute of Marine Sciences, National Research Council, Venice, Italy 30122; § School of Mathematics and Statistics, 3526 University of Glasgow, Glasgow, United Kingdom G12 8TA

**Keywords:** transitional waters, Venice Lagoon, SHYFEM, Landsat 8, TACT, thermal plumes, surface water temperature

## Abstract

Monitoring surface water temperature (SWT) in transitional environments remains challenging due to the interplay of natural and anthropogenic processes, which introduce greater complexity than in open-ocean systems. This study evaluates four SWT products for their ability to capture temperature dynamics in the Venice Lagoon, a well-monitored coastal system. The assessment included (1) output from the hydrodynamic model SHYFEM (System of Hydrodynamic Finite Element Module), (2) a satellite-based Level 4 product from the European Space Agency Climate Change Initiative (ESA CCI), (3) the Landsat 8 Level 2 standard thermal product, and (4) Landsat 8 Level 1 data processed using the Thermal Atmospheric Correction Tool (TACT). Validation against in situ observations indicated that SHYFEM and TACT showed lower bias (−0.48 °C and −0.30 °C, respectively) and RMSE (∼1.2 °C) than the other products. SHYFEM effectively reproduced intra-annual SWT trends with comprehensive temporal coverage, while TACT captured fine-scale spatial features, including thermal anomalies from industrial discharges. Building on this, an integrated product combining SHYFEM and TACT was developed, providing a more accurate and coherent representation of spatiotemporal SWT dynamics. This transferable framework advances understanding of thermal variability in transitional waters and has potential to support ecosystem management and climate adaptation strategies.

## Graphical abstract

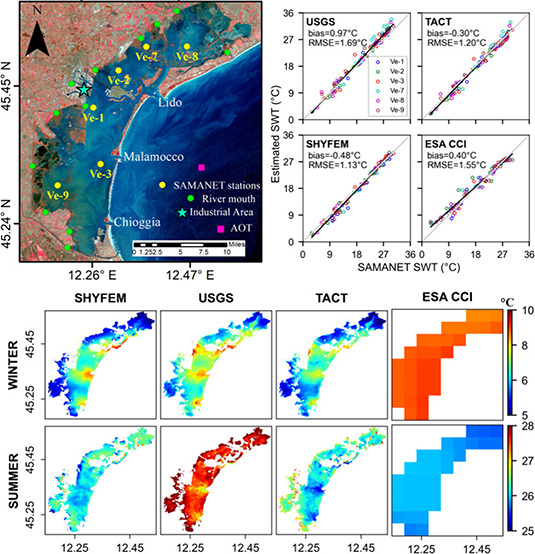

## 1 Introduction

SWT is a critical Essential Climate Variable (ECV) that drives ocean-atmosphere interactions, shaping the hydrological cycle, biogeochemical processes, and marine ecosystems.^1–5^ Transitional environments, such as coastal zones, estuaries, and lagoons, represent dynamic interfaces between land and sea and are highly sensitive to natural variations and human activities.^6^ In the open ocean, temperature is mainly governed by solar heating, air-sea heat exchange, wind patterns, and ocean currents.^7^ However, at the land–sea interface, water temperature variability can be influenced by a range of additional factors, including river inflows, tidal currents, groundwater discharge, and geographical characteristics like location and bathymetry. Human activities such as urbanization, industrial wastewater, agricultural runoff, and coastal development further amplify this complexity.^8–11^

Given the complex drivers of SWT in transitional environments, effective monitoring is crucial for capturing fine-scale variability and supporting climate and ecosystem management efforts.^12^ Satellite-based observations and innovations in computational techniques^13,14^ have revolutionized SWT monitoring over the past four decades, providing continuous measurements through thermal infrared sensors.^15^ These observations have enabled the creation of long-term, high-quality global SWT time series data sets, under the Group for High Resolution Sea Surface Temperature (GHRSST) framework, critical for understanding oceanic and climatic processes.^15–20^ Such global data sets are effective in offshore areas, where temperature spatial variability is relatively smooth, making them ideal for climate-related applications.^21^ However, in transitional systems, the high spatial variability driven by hydrological inputs, geographical features, and human activities reduces the accuracy of the data set.^22,23^ Coastal dynamics require sensors with high spatial resolution to capture fine-scale variability. The Landsat 8 Thermal Infrared Sensor (TIRS) provides SWT estimates at a native resolution of 100 m, well-suited for monitoring lagoons and inland water bodies.^24–26^ This sensor offers critical insights into SWT dynamics and is effective for detecting thermal pollution,^27,28^ mapping river plumes,^29^ and identifying localized temperature anomalies.^30^ However, such sensors still face challenges from cloud cover and data completeness, underscoring the ongoing need for complementary approaches to comprehensively understand the dynamics of transitional water bodies.^31^

Over the past few decades, a range of open-source and community-supported hydrodynamic models have been developed for ocean and coastal studies. These models offer a cost-effective approach to simulating real-world systems, providing continuous spatial and temporal coverage that enables deeper insights into the dynamics of SWT.^13,32–34^ Deterministic models like SHYFEM (System of HydrodYnamic Finite Element Module), developed by the Institute of Marine Sciences (ISMAR) of the Italian National Research Council, employs unstructured finite element grids, which enable high-resolution modeling of irregular geometries, narrow channels, and shallow water bodies.^35^ It has been widely applied to many coastal lagoons^36^ to simulate parameters such as water levels, temperature, and salinity, and has been used to assess the impacts of storm surges and future sea-level rise scenarios.^37,38^ However, hydrodynamic models face limitations in representing air-sea and bottom-sea processes, as well as land-sea interactions and freshwater inputs, while high computational demands further constrain their accuracy and broader applicability.^39^

Integrating satellite-based and hydrodynamic model-based SWT offers a promising approach to overcoming the spatial and temporal limitations of traditional monitoring techniques in complex transitional environments.^40^ This synergy reduces spatial and temporal data gaps, enhances simulation accuracy, and opens avenues for advanced predictive applications by leveraging emerging machine learning capabilities.^41^ The resulting high-resolution data sets improve the understanding of the dynamics of coastal and lagoon systems and support evidence-based management strategies from short-term to long-term and across regional to global scales.^42^

This study aims to demonstrate the significance of integrating satellite-based observations with hydrodynamic model outputs to better understand SWT dynamics in a complex transitional environment. The Venice Lagoon, a well-monitored and climatically vulnerable coastal system,^43–45^ was chosen as the study site due to its complex geomorphology and exposure to both natural and anthropogenic pressures. Four SWT products were evaluated: output from the SHYFEM hydrodynamic model, and three satellite-based data sets comprising the ESA CCI Level 4 product, the Landsat 8 Level 2 standard thermal product, and Landsat 8 Level 1 data processed with the Thermal Atmospheric Correction Tool (TACT). A detailed description of each product and the criteria for their selection are provided in the Supporting Information (section S1). The goal is to test the applicability of each SWT product at the lagoon scale, evaluating whether they effectively capture temperature dynamics while aligning with in situ observations. Finally, the integration of model and satellite products is explored as a pathway to improve accuracy and generate a more coherent representation of spatiotemporal SWT variability in transitional waters.

## 2 Materials and Methods

### 2.1 Study Site

The Venice Lagoon, a UNESCO heritage site rich in cultural history spanning over 1000 years, is the largest lagoon in the Mediterranean. It features three inlets: Lido, Malamocco, and Chioggia, connecting with the Adriatic Sea (Figure 1). The Venice lagoon, spanning 550 km^2^, encompasses over 75% of shallow depths less than 2 m, with an average depth of 1.2 m.^46^ Historical human influences, like river redirection and channel dredging, have altered its morphology, leading to challenges such as erosion and water quality degradation.^47^ Climate change has further intensified these pressures, with surface waters warming by more than 1 °C over the past three decades and a marked increase in the frequency and severity of marine heat waves. In parallel, sea level rise has driven major management interventions. The MoSE (Modulo Sperimentale Elettromeccanico) barriers were constructed at the three inlets to protect Venice from flooding.^48^ They have been operational since October 2020 ^49^ and were deployed more than 100 times by April 2025 (https://www.mosevenezia.eu/?lang=en). Barrier closures are becoming increasingly frequent,^50^ and their operation is projected to reduce winter water exchange through the inlets, with significant implications for lagoon thermal dynamics.^48^ To monitor these impacts, the lagoon is supported by dense in situ observational networks that provide high-frequency, spatially distributed measurements of key variables, including water temperature, sea level, and meteorological conditions.^43,48^ This complex setting, shaped by multiple interacting pressures, makes the Venice Lagoon a critical example of a transitional environment where effective management policies are required for ecosystem preservation.

**Figure 1:**
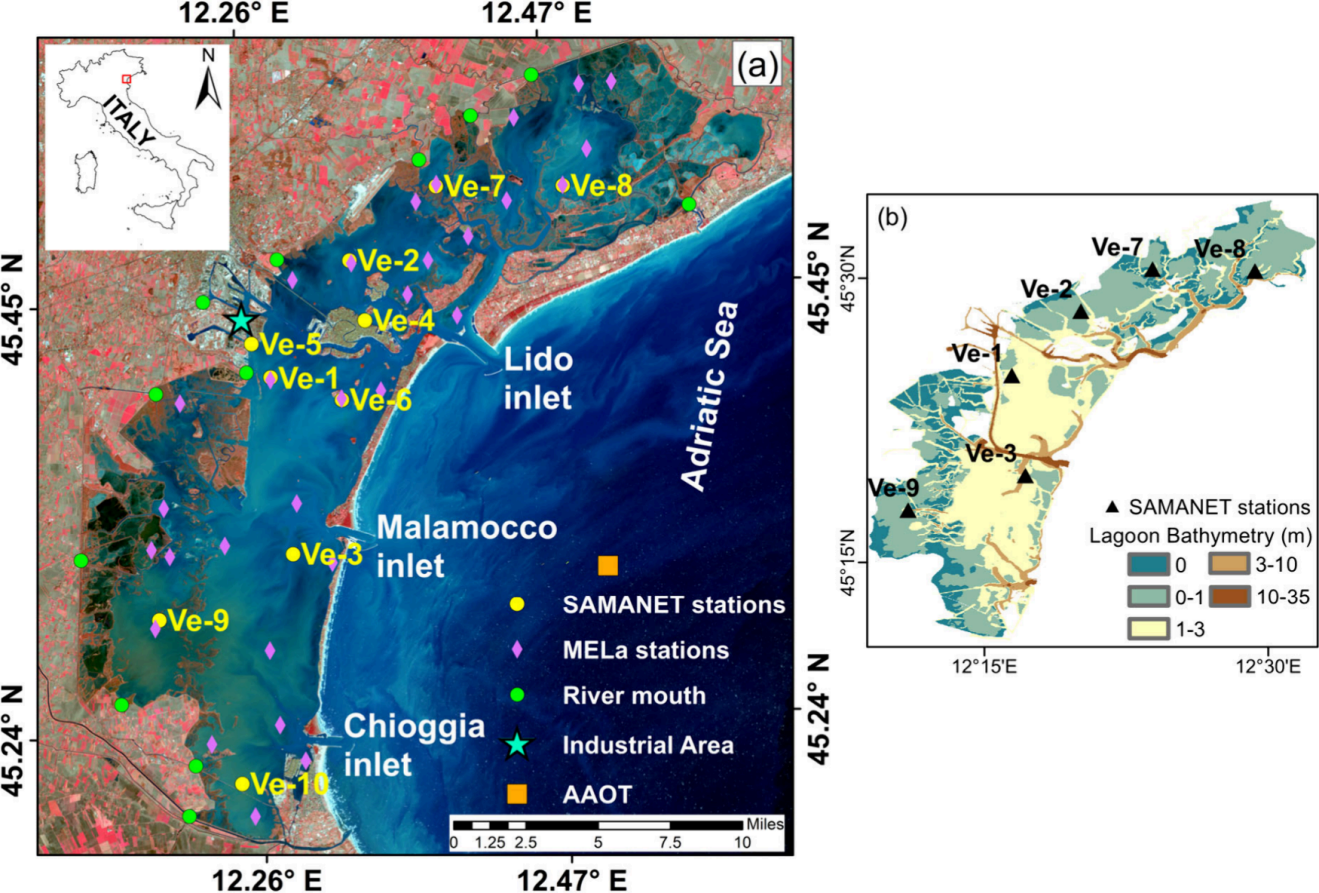
(a) Map of the Venice Lagoon showing the SAMANET monitoring stations (yellow dots), ARPAV monitoring network (pink diamonds), river mouths (green dots), the industrial area (cyan star), and the Acqua Alta Oceanographic Tower (AAOT, orange square). (b) Bathymetric map of the lagoon with the SAMANET stations (black triangles, 6 sites used for the analysis), overlaid on depth classes from tidal flats (0–1 m) to deeper channels and inlets (up to 35 m).

### 2.2 Hydrodynamic Modeling

SHYFEM, a finite element 3D hydrodynamic model was implemented across the Venice Lagoon spatial domain. The model resolves the shallow water equations, incorporating hydrostatic and Boussinesq approximations. It employs a finite element approach for horizontal spatial discretization, and time integration is addressed through a semi-implicit algorithm.^35^ The model simulates hydrodynamics and the advection-diffusion processes responsible for heat transport in the water column. The model incorporates heat exchange with the atmosphere through solar radiation, longwave radiation, sensible heat flux, and latent heat flux.^51,52^

The hydrodynamic simulations were conducted using the calibrated SHYFEM model, with an unstructured finite element grid. The spatial grid consisted of 53,463 nodes and 100,308 triangular elements, covering the Venice Lagoon and a small portion of the Adriatic Sea, extending 20 km offshore. The finite element module employs variable element sizes, ranging from meter-scale inside the lagoon to kilometre-scale in the offshore regions of the Adriatic Sea, allowing it to adapt to the basin’s complex geometry. Vertically, the model operated in a zeta-layer configuration, employing 13 vertical layers with increasing thickness with depth. The top six layers were 1 m each, while the subsequent layers progressively increased, reaching 4 m in the deepest layers, with a maximum depth of 40 m.

Simulations were carried out for the entire year 2019 using the 3D version of the model in baroclinic mode, enabling the computation of baroclinic pressure gradients resulting from temperature and salinity variations. The model simulations were forced using real-time meteo-marine data from 2019, including tides, wind, rainfall, and heat fluxes. Initial conditions at the seaward open boundary were defined based on field measurements collected within the lagoon, along the coastal area, and at the Acqua Alta Oceanographic Tower (AAOT) during the simulation period. Real-time observations of tidal level, velocity, temperature, and salinity at the Adriatic Sea–Lagoon interface were used as boundary conditions. The simulations were run with a time step of 10 s. River discharges into the lagoon, recorded at hourly intervals through sensor networks operated by ARPAV (Regional Environmental Protection Agency of Veneto) and the Consorzio di Bonifica (Land Reclamation Consortium of Veneto), were incorporated into the model setup (green dots in Figure 1 indicate the river mouths). The model was configured to provide SWT outputs at hourly intervals, representing temperatures at approximately 0.5 m depth. More details on the hydrodynamic model configuration are outlined in.^36,51^

### 2.3 Earth Observation of SWT

Daily SWT data from the ESA CCI v3.0 data set, which combines observations from multiple infrared and microwave sensors, were analyzed in this study. The gap-filled, interpolated, Level 4 analysis product provides daily mean SWT at a depth of 0.2 m referenced to 10:30 local mean solar time, with a spatial resolution of 0.05°.^16^ For this study, daily SWT maps for the Venice Lagoon during 2019 were collected through surftemp.net. More details on the data are provided in the studies by.^16^ This product will hereafter be referred to as ESA CCI.

The Thermal Infrared Sensor (TIRS) and Operational Land Imager (OLI) on board Landsat 8, with OLI providing 30 m resolution and TIRS offering 100 m native resolution resampled to 30 m, were also used. The Collection 2 Level 1 (C2L1) top-of-atmosphere radiance and the Collection 2 Level 2 (C2L2) standard surface temperature science product were obtained through the USGS Earth Explorer platform (USGS - EarthExplorer).^53^ The C2L2 data set is generated from C2L1 employing the MODTRAN radiative transfer model, facilitating the computation of crucial atmospheric parameters such as upwelling (L_u_) and downwelling (L_d_) radiances and transmittance (τ).^54^ Using these derived atmospheric parameters alongside a constant emissivity value (0.988) assigned to water pixels, the total radiance measured in band 10 (approximately 10 μm) of the C2L1 TIRS data was transformed into skin SWT, which represents the temperature of the uppermost micrometres of the water. This product will hereafter be referred to as USGS.

The C2L1 TIRS data were further processed using the Thermal Atmospheric Correction Tool (TACT) developed for Landsat and implemented in ACOLITE, an open-source software commonly used for atmospheric correction of satellite images.^55,56^ TACT integrates libRadtran, an open-source radiative transfer model, to compute atmospheric parameters (L_u_, L_d_, τ) using atmospheric profiles of relative humidity and temperature derived from ERA5 climate data (0.25° resolution) provided by the European Centre for Medium-Range Weather Forecasts (ECMWF). It employs an emissivity value of 0.9926 for deriving SWT.^57^ This product will hereafter be referred to as TACT. The Level 1 OLI top-of-atmosphere reflectance data were atmospherically corrected using ACOLITE (with default settings) to derive Level 2 surface reflectance products. Both OLI reflectance data (Level 1 and Level 2) were used for the quality control of matchup data, which were then used for the comparative studies explained in the section 2.5.

### 2.4 In Situ SWT

The Interregional Public Works Department for Veneto, Trentino Alto Adige, and Friuli Venezia Giulia operates a real-time monitoring network known as SAMANET (Sezione Anti-inquinamento Magistrato alle Acque – NET), which comprises 10 stations distributed across the Venice Lagoon (see Figure 1a).^58^ Each station is equipped with multiparametric probes measuring seven water quality parameters, including SWT, at 30 min intervals at a depth of about 1 m.^59^ In situ SWT data (hereafter referred to as SAMANET) measured at six stations (Ve-1, Ve-2, Ve-3, Ve-7, Ve-8, and Ve-9) were available for 2019 and were used to evaluate the accuracy of SHYFEM and Earth Observation products. These stations span the northern, central, and southern sectors of the lagoon, capturing marine, freshwater-influenced, and industrial zones. Additional SWT observations from the ARPAV (Regional Agency for Environmental Prevention and Protection of Veneto) monitoring program were also used as an independent reference to validate SHYFEM outputs (see section S2 of Supporting Information for details). The SWT data sets used in this study are summarized in Table 1.

**Table 1:** Summary of SWT Data Sets Used in This Study

SWT products	Spatial Resolution	Temporal Resolution	Brief Description	Source
SHYFEM	Variable (grid-based)	hourly	3D hydrodynamic model providing high-resolution simulations of SWT, driven by atmospheric and boundary forcing	https://github.com/SHYFEM-model/shyfem
ESA CCI	0.05° (∼5 km)	daily	Gap-filled climate data record of SWT, derived from multiple satellite missions	https://surftemp.net/
USGS	30 m	16 days	Level 2 standard product from Landsat 8 Thermal Infrared Sensor (TIRS)	https://earthexplorer.usgs.gov/
TACT	30 m	16 days	Landsat 8 Level 1 TIRS data processed with TACT	https://earthexplorer.usgs.gov/, https://github.com/acolite/tact
SAMANET	Point-based	30 min	Real-time observations using multiparametric probes, used for validation	http://provveditoratovenezia.mit.gov.it/
ARPAV	Point-based	Seasonal (4 times per year)	CTD campaigns, used for validation	https://www.arpa.veneto.it/

### 2.5 Assessing Performance of Model and Satellite Data

The comparison was conducted in two parts: (1) at discrete locations and (2) over a spatial scale encompassing the entire lagoon. For the discrete locations, SWT values from SHYFEM and SAMANET stations at 10:00 UTC were used, corresponding to the acquisition time of Landsat 8 images (between 9:50 and 10:00 UTC) over the Venice Lagoon. Gap-free SHYFEM and ESA CCI estimates were extracted at the six SAMANET locations, using model nodes and the coinciding 1 × 1 pixel, respectively. For the Landsat 8 products, the quality control procedure described by^57^ was applied to mask land, clouds, high glint, and floating objects or vegetation. Unlike the 5 × 5 window used by,^57^ SWT values were extracted from the closest coinciding pixels to minimize land adjacency effects, as some SAMANET stations are located in narrow tidal channels surrounded by marsh areas. Though the SWT estimates correspond to different measurement depths, the shallow, well-mixed conditions of the Venice Lagoon minimize vertical temperature differences,^60^ enabling direct comparison among the selected products. To assess the accuracy and robustness of satellite- and model-based temperature estimates against in situ measurements from the SAMANET stations, the following statistical metrics were computed: coefficient of determination (R^2^), bias, root-mean-square error (RMSE), and mean absolute percentage error (MAPE).

1 $$R^{2}=\frac{\overset{n}{\underset{i=0}{\sum }}\left(X_{i}-\mathrm{X̅}\right)\left(Y_{i}-\mathrm{Y̅}\right)}{\sqrt{\overset{n}{\underset{i=0}{\sum }}{\left(X_{i}-\mathrm{X̅}\right)}^{2}}\sqrt{\overset{n}{\underset{i=0}{\sum }}{\left(Y_{i}-\mathrm{Y̅}\right)}^{2}}}$$

2 $$bias=\overset{n}{\underset{i=0}{\sum }}\frac{X_{i}-Y_{i}}{n}$$

3 $$RMSE=\sqrt{\overset{n}{\underset{i=0}{\sum }}\frac{{\left(X_{i}-Y_{i}\right)}^{2}}{n}}$$

4 $$MAPE=100\times \frac{1}{n}\overset{n}{\underset{i=0}{\sum }}\left|\frac{X_{i}-Y_{i}}{Y_{i}}\right|$$

where X denotes SWT estimates from SHYFEM or satellite-based products (ESA CCI, USGS, and TACT), Y is the corresponding SAMANET observation, and n is the number of observations where both X and Y are available. ARPAV data was also used to assess the performance of SHYFEM using the above four metrics.

For spatial analysis, 18 cloud-free Landsat 8 TIRS-derived SWT products (both USGS and TACT) were used. To facilitate direct comparison with Landsat 8 data, SHYFEM estimates at 10:00 UTC on corresponding image dates were interpolated from varying grid sizes onto a uniform grid of 0.0003° in latitude and longitude, matching the satellite’s spatial resolution. The spatial maps of the ESA CCI products were maintained at their native resolution. To assess seasonal variability, composites were generated for each product by averaging SWT values over winter (December–February), spring (March–May), summer (June–August), and autumn (September–November), using the same spatial mask as applied to the Landsat 8 products. Additionally, spatial agreement between SHYFEM and the Landsat 8-derived SWT products was assessed, and the corresponding statistics are reported in the Supporting Information (section S3).

### 2.6 Spatial Fusion of SHYFEM and TACT SWT Fields

To produce a spatially enhanced SWT field, we developed a fusion strategy that integrates SHYFEM-derived estimates with satellite-based TACT observations. The goal is to preserve the broad-scale physical consistency of SHYFEM while incorporating the fine-scale spatial variability captured by TACT, particularly in regions where TACT has demonstrated superior accuracy. This fusion approach leverages the complementary strengths of both data sets to yield an SST product that is both physically coherent and spatially detailed.

The fusion method follows a weighted blending framework, where TACT corrections are applied locally to the SHYFEM field based on their spatial proximity to trusted validation points (e.g., stations Ve-1 and Ve-2). Gaussian weighting functions are used to assign influence based on distance from these trusted stations. At each spatial location (x, y), the TACT weight for station i is given by

5 $$\omega_{i}\left(x,y\right)=\exp\left(-\frac{{\left(x-x_{i}\right)}^{2}+{\left(y-y_{i}\right)}^{2}}{2\sigma^{2}}\right)$$

where (x_i_, y_i_) denotes the location of station i, and σ defines the spatial scale of influence (set to 0.03 degrees, approximately 3 km). The fused SST at each grid point is then computed as a convex combination of TACT and SHYFEM values:

6 $$SWT_{FUSED}\left(x,y\right)=\frac{\underset{i}{\sum }\omega_{i}\left(x,y\right)T\left(x,y\right)+\left(1-W_{T}\left(x,y\right)\right)S\left(x,y\right)}{\underset{i}{\sum }\omega_{i}\left(x,y\right)+\left(1-W_{T}\left(x,y\right)\right)}$$

where *T*(*x*,*y*) is the TACT SST estimate, *S*(*x*,*y*) is the SHYFEM SST estimate, and *W*_*T*_(*x*,*y*) = *min*(1,∑_*i*_ω_i_(*x*,*y*)) ensures that the total weight assigned to TACT does not exceed unity. This formulation enforces a smooth spatial transition between the two sources, emphasizing TACT in regions of high confidence while reverting to SHYFEM where satellite data are less reliable. The normalization guarantees a physically meaningful blend, reducing discontinuities and artifacts at interface zones.

## 3 Results

### 3.1 Comparison between SAMANET and SHYFEM SWT

The observed intra-annual SWT across the SAMANET stations (Figure 2a) averaged around 18 °C, with a winter minimum of 2.16 °C and a summer maximum of 31.65 °C. Winter temperatures ranged from 2.5 to 10 °C, followed by a gradual increase from 10 to 24 °C during spring. Summer temperatures spanned from 25 to 31.65 °C, with notable peaks in late June and July, coinciding with strong Marine Heat Wave (MHW) events observed in the northwest Mediterranean.^61^ During the winter months, temperature measurements between the six SAMANET stations demonstrated pronounced variability, with differences between minimum and maximum SWT frequently exceeding 4 °C. Particularly, Station Ve-1, located near an industrial area, recorded notably higher temperatures during winter and early spring, with differences ranging from 3 to 6 °C. Followed by winter, the lagoon gradually warmed during spring and summer, reaching its peak temperature in late July. During this period (late spring and summer), spatial variability was minimal, with temperatures across the stations typically differing by at most 2 °C. Station Ve-3, situated near the Malamocco Inlet, recorded lower SWT compared to other stations during June and July, likely influenced by tidal exchange with the Adriatic Sea. During the autumn months, the variability in SWT closely resembled that observed in spring and winter, following a decreasing trend from 26 to 11 °C. The SWT time series presents missing data at some stations due to maintenance works, like e.g. Ve-3, Ve-7, and Ve-9.

**Figure 2:**
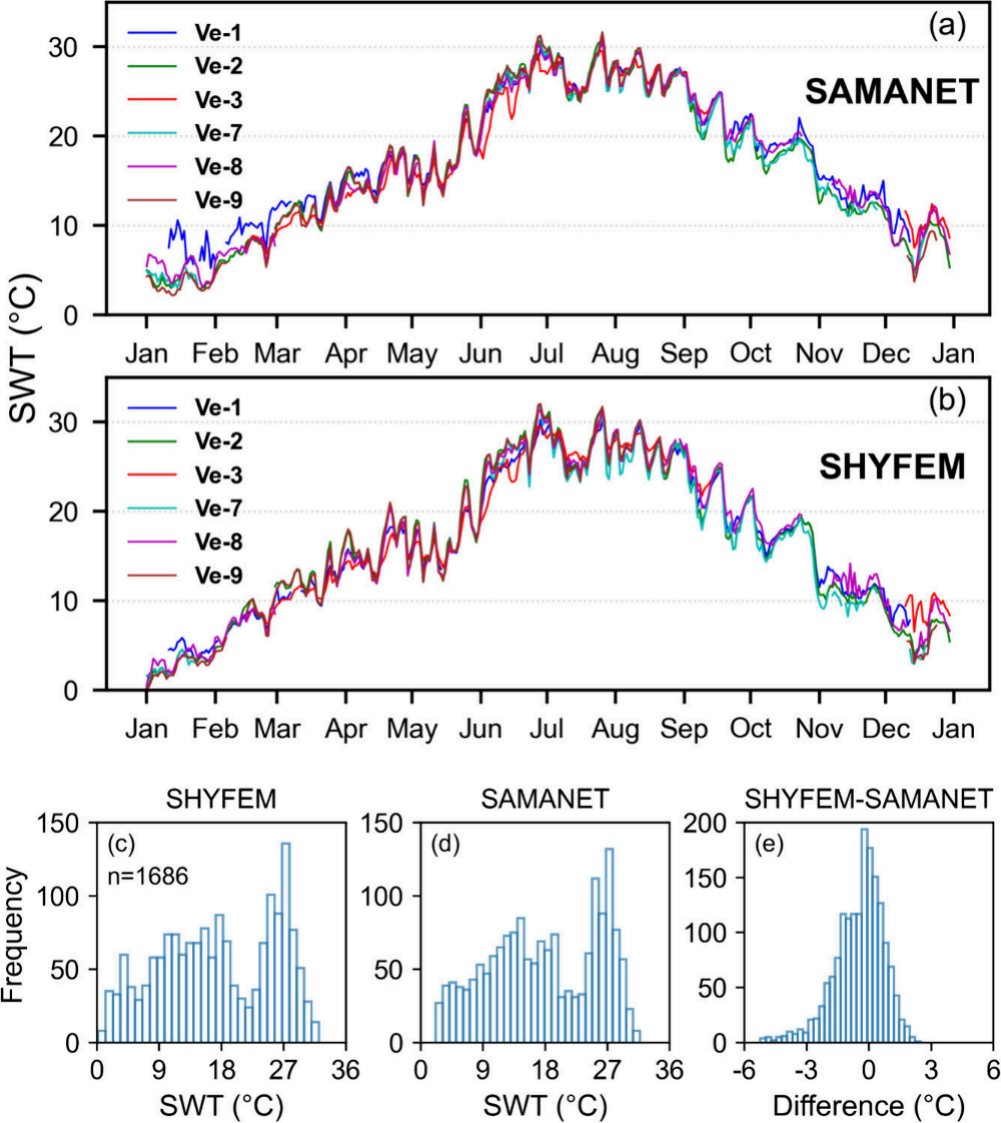
Intra-annual variation of SWT from SAMANET (a) and SHYFEM (b) at six locations within Venice Lagoon for the year 2019. Bottom panels show the histogram of SWT from SHYFEM (c), SAMANET (d), and corresponding difference (e).

The hydrodynamic model SHYFEM closely replicated the temporal temperature trends observed by SAMANET (Figure 2b), with R^2^ values exceeding 0.98 for all stations, demonstrating strong agreement with the observed variations. The model effectively captured the spatial variations in SWT across the lagoon, showing good alignment with the SAMANET probe data. The SWT distribution from SHYFEM ranged from 0 to 32 °C, with a peak around 27 °C (Figure 2c). The SAMANET data exhibited a similar distribution shape but with minimum temperatures starting from approximately 2 °C (Figure 2d). This behavior was reflected in the distribution of the SHYFEM-SAMANET difference values, which, while strongly clustered within ± 1 °C, exhibited an extended tail toward negative values (Figure 2e).

SHYFEM tended to underestimate SWT by 1–2 °C during the winter months. Notably, station Ve-1 showed a greater underestimation, a trend that persisted through all seasons except summer. The RMSE (1.56 °C) and bias (−1.14 °C) for Ve-1 were relatively higher compared to other stations (Table 2). However, SHYFEM demonstrated strong overall performance in estimating SWT across all six stations, effectively capturing both spatial and temporal variability with minimal bias (−0.48 °C), RMSE (1.28 °C), and MAPE (8.45%).

**Table 2:** Performance Metrics (Bias, RMSE, and MAPE) for SWT Estimates from SHYFEM and ESA CCI, Compared against SAMANET Observations at Individual Stations across the Venice Lagoon for the Year 2019^a^

		bias (°C)		RMSE (°C)		MAPE (%)	
Stations	SAMANET Observations (n)	SHYFEM	ESA CCI	SHYFEM	ESA CCI	SHYFEM	ESA CCI
Ve-1	324	–1.14	–0.35	1.56	1.47	9.16	8.26
Ve-2	358	–0.32	0.62	1.21	1.94	8.8	16.08
Ve-3	228	0.11	0.15	0.73	0.9	3.26	4.02
Ve-7	205	–0.91	0.81	1.43	1.9	9.07	15.56
Ve-8	326	–0.45	0.18	1.29	1.44	8.84	10.44
Ve-9	245	–0.02	0.32	1.05	2.02	8.89	23.08
**All stations**	**1686**	**–0.48**	**0.26**	**1.28**	**1.66**	**8.45**	**12.81**

a The column “SAMANET observations (n)” indicates the number of days with simultaneous availability of SAMANET observations and corresponding SHYFEM and ESA CCI data. Values in bold highlight the overall performance metrics across all stations.

### 3.2 ESA CCI SWT

The ESA CCI product captured the seasonal and intra-annual trend of SWT. However, the coarser spatial resolution of the ESA CCI (∼ 5 km) product resulted in uniform temperature estimates across all stations for a given day, lacking the ability to resolve spatial variation within the lagoon (Figure 3a). This highlights the importance of high spatial resolution sensors for a more detailed understanding of coastal and inland water bodies. The distribution of SWT from ESA CCI confined between 5 and 29 °C, as shown in Figure 3b. The difference between ESA CCI and SAMANET measurements exhibited variability predominantly within ± 3 °C (Figure 3c), with a noticeable skew toward positive values. This trend arose from an underestimation of summer temperatures (negative tail) and an overestimation during the other seasons (positive tail). Statistical evaluation (Table 2) showed that ESA CCI exhibited a positive bias (0.26 °C), higher RMSE (1.66 °C), and notably higher MAPE (12.81%), indicating greater variability and less accuracy compared to SHYFEM. Overall, despite its coarse spatial resolution, the ESA CCI product effectively reproduced temporal trends and closely reflected the surface temperature of the Adriatic Sea, as observed from the agreement with SAMANET observations at station Ve-3 located near the inlet.

**Figure 3:**
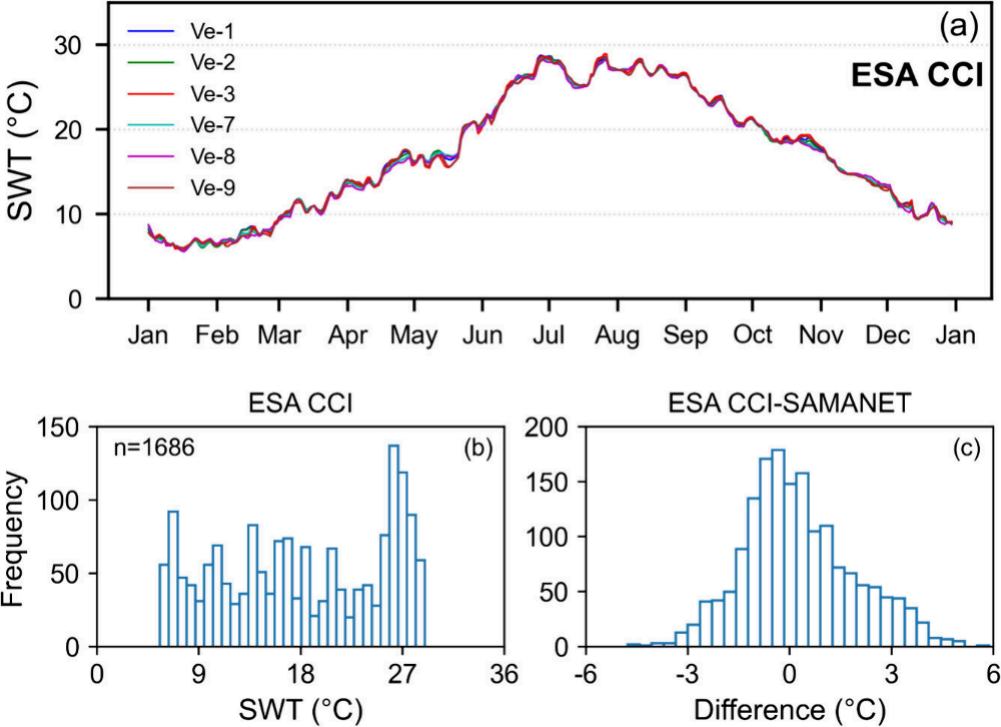
SWT from Level 4 analysis ESA CCI product. (a) Time series of SWT. (b) Histogram of SWT distribution. (c) Histogram of difference between ESA CCI estimates and SAMANET observations.

### 3.3 Validation of SWT Products

A total of 101 matchups were selected following quality control, supporting the validation of SWT estimates from Landsat 8 products. For consistency, SHYFEM and ESA CCI performances were also evaluated for the corresponding matchup data set. Distinct trends were observed for each product in comparison with the SAMANET data. The USGS product (Figure 4a) overestimated SWT, with positive bias (0.97 °C) and higher RMSE (1.69 °C), and this overestimation was pronounced during summer. In contrast, the TACT-derived SWT values exhibited a remarkable alignment on a 1:1 scale with SAMANET measurements, showcasing a high level of consistency (Figure 4b). However, at Ve-7 (cyan markers in Figure 4b), located close to marshland, TACT consistently overestimated summer SWT, likely due to the influence of warmer surrounding land surfaces on the retrievals. SHYFEM marginally underestimated temperatures in the lower ranges (below 16 °C, Figure 4c), while the ESA CCI product showed overestimation at lower SWT and underestimation at higher SWT (Figure 4d). Overall, the R^2^ exceeded 0.96 for all products. However, the RMSE and MAPE were lowest for TACT (1.2 °C and 6.79%) and SHYFEM (1.13 °C and 6.02%) compared to the standard USGS and ESA CCI products. Additional validation against ARPAV monitoring data demonstrated the ability of SHYFEM to capture the spatiotemporal temperature dynamics across the lagoon (Figure S1 in Supporting Information).

**Figure 4:**
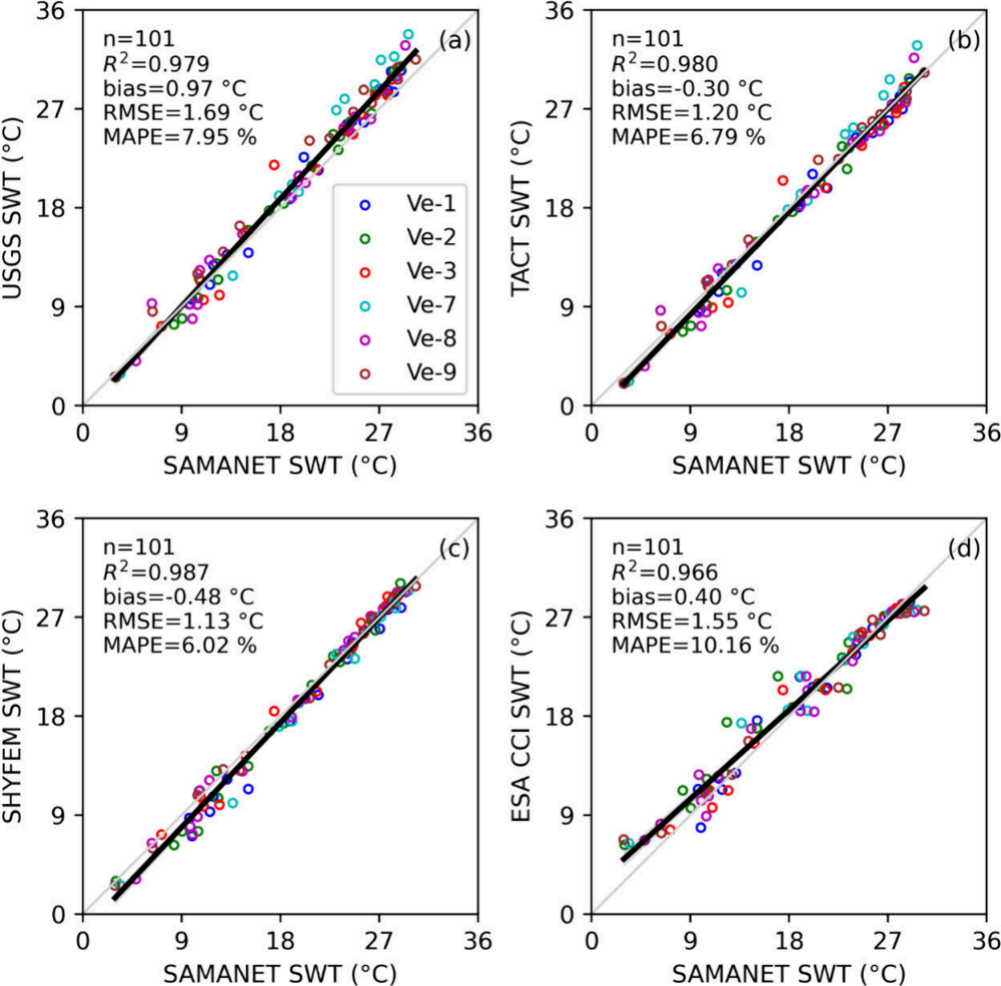
Scatterplots comparing SWT estimates from Landsat 8 derived products, SHYFEM, and ESA CCI with SAMANET observations at six stations (Ve-1, Ve-2, Ve-3, Ve-7, Ve-8, and Ve-9), based on the Landsat 8 matchups (*n* = 101). Panels represent comparisons for (a) USGS, (b) TACT, (c) SHYFEM, and (d) ESA CCI. The light gray diagonal line indicates the 1:1 line, while the solid black line represents the linear regression fit. Statistical metrics (R^2^, bias, RMSE, and MAPE) were calculated from paired observed and estimated SWT values.

### 3.4 Spatiotemporal Analysis

With SHYFEM demonstrating strong agreement with SAMANET observations and providing continuous spatiotemporal coverage, its estimates were used as a reference for the spatial analysis. The high spatial resolution seasonal SWT maps from SHYFEM, the USGS standard product, and the TACT product captured detailed temperature gradients within the lagoon (Figure 5). During winter, SHYFEM, USGS, and TACT maps consistently showed lower SWT across the lagoon, ranging from approximately 4 to 10 °C. Higher values were observed in the inlet channels (black arrows in winter map from SHYFEM, Figure 5), attributed to tidal exchanges with the warmer Adriatic Sea, while lower temperatures in the northern lagoon were likely influenced by river inputs and shallow bathymetry. In spring, SHYFEM and TACT maps showed lagoon-wide temperatures between 11 and 14 °C, with warmer waters observed near the land boundaries. The USGS product depicted a similar spatial pattern but tended to overestimate SWT, with values often exceeding 14 °C. Summer exhibited the highest SWT values, with SHYFEM and TACT ranging from 25 to 28 °C. The USGS product continued to show elevated SWT values, with widespread areas exceeding 28 °C. Notably, relatively cooler temperatures were observed in the inlet channels compared to the surrounding lagoon, visible in the USGS and TACT products. In autumn, a cooling trend was observed across all products. SHYFEM and TACT showed spatial gradients between 16 and 22 °C, with spatial variability patterns similar to those in winter. Given the absence of ground truth temperature data for regions beyond the SAMANET stations, quantifying the nature of discrepancies across the entire lagoon remains challenging. In contrast to the other products, the ESA CCI SWT data set exhibited limited spatial variability within the lagoon due to its coarser resolution and showed higher temperatures in winter and lower temperatures in summer relative to SHYFEM estimates. Overall, SHYFEM and TACT products captured the spatial heterogeneity and seasonal transitions of SWT more effectively than ESA CCI and USGS, the latter of which tended to overestimate temperatures. This was further confirmed through quantitative spatial comparison (see section S3 in Supporting Information).

**Figure 5:**
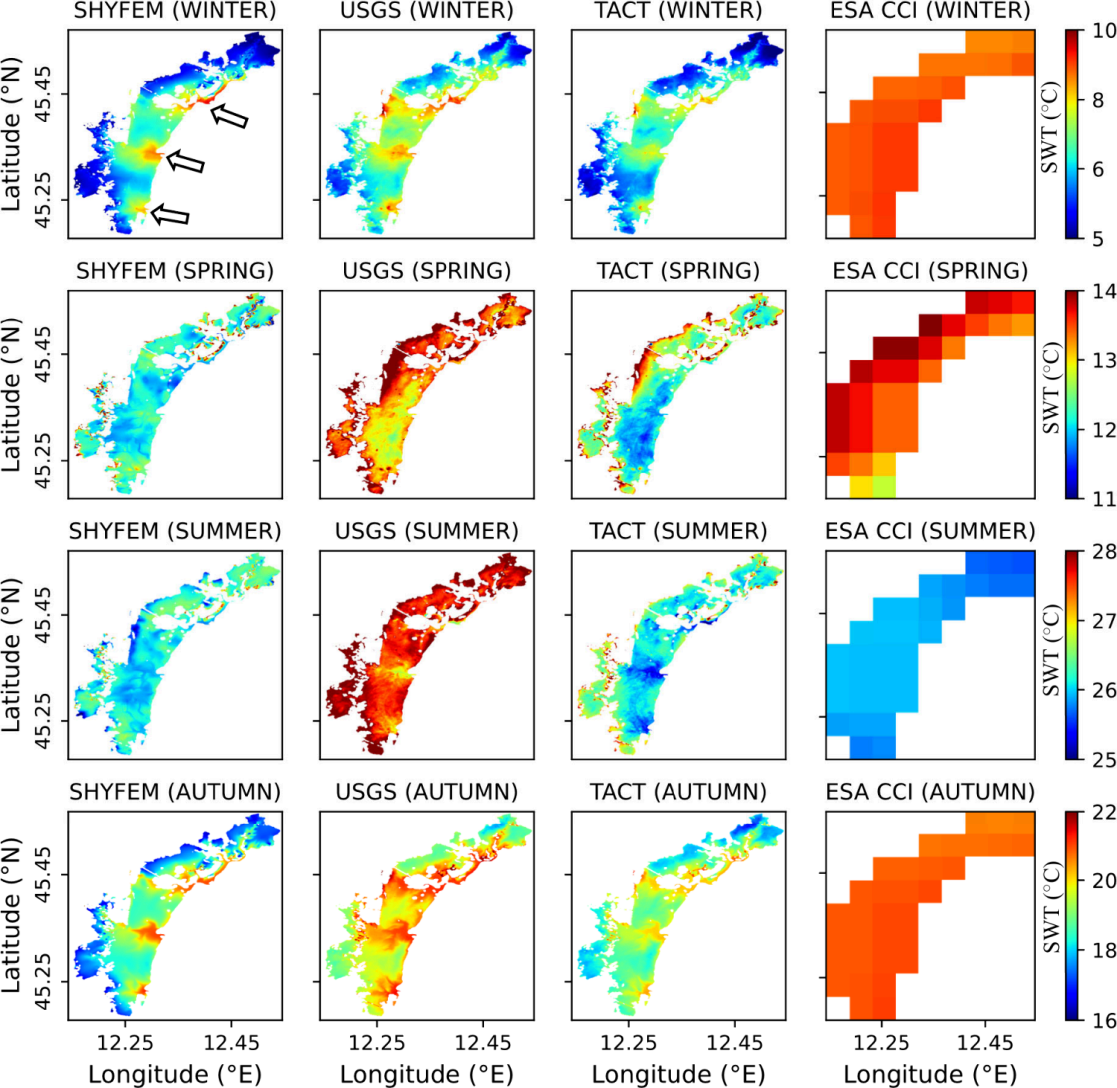
Spatial maps of surface water temperature (SWT) from SHYFEM, USGS, TACT, and ESA CCI (columns 1–4, respectively) for winter, spring, summer, and autumn (rows 1–4, respectively). Seasonal composites were generated using 5 images from winter, 3 from spring, 5 from summer, and 5 from autumn (based on the availability of cloud-free scenes of Landsat 8). Three arrows in the top-left panel (first row, first column) indicate the three inlets (Lido, Malamocco, and Chioggia).

### 3.5 Anthropogenic Thermal Influences

Beyond the broad seasonal and spatial variability of lagoon temperature, localized industrial discharges exert a pronounced influence on SWT in specific areas of the Venice Lagoon. Figure 6a–d presents SWT maps from SHYFEM and TACT for 24 January 2019 (a, b) and 26 December 2019 (c, d). The maps highlight two locations of warm water injection into the lagoon: Port Marghera (black dot) and the thermal power plant outlet at Fusina (blue dot), with SAMANET station Ve-1 (brown dot) located within the highlighted pink circle. The high-resolution TACT product effectively captured the spatial extent of the thermal anomaly, clearly delineating thermal plumes on both days. In contrast, SHYFEM outputs indicated only modest warming in these regions, with the localized plumes not distinctly resolved, which contributed to higher errors at Ve-1 (Table 2). TACT observed anomalies exceeding 5 °C near the power plant outlet (blue dot) relative to surrounding areas, indicating strong localized warming and potential microclimatic effects.

**Figure 6:**
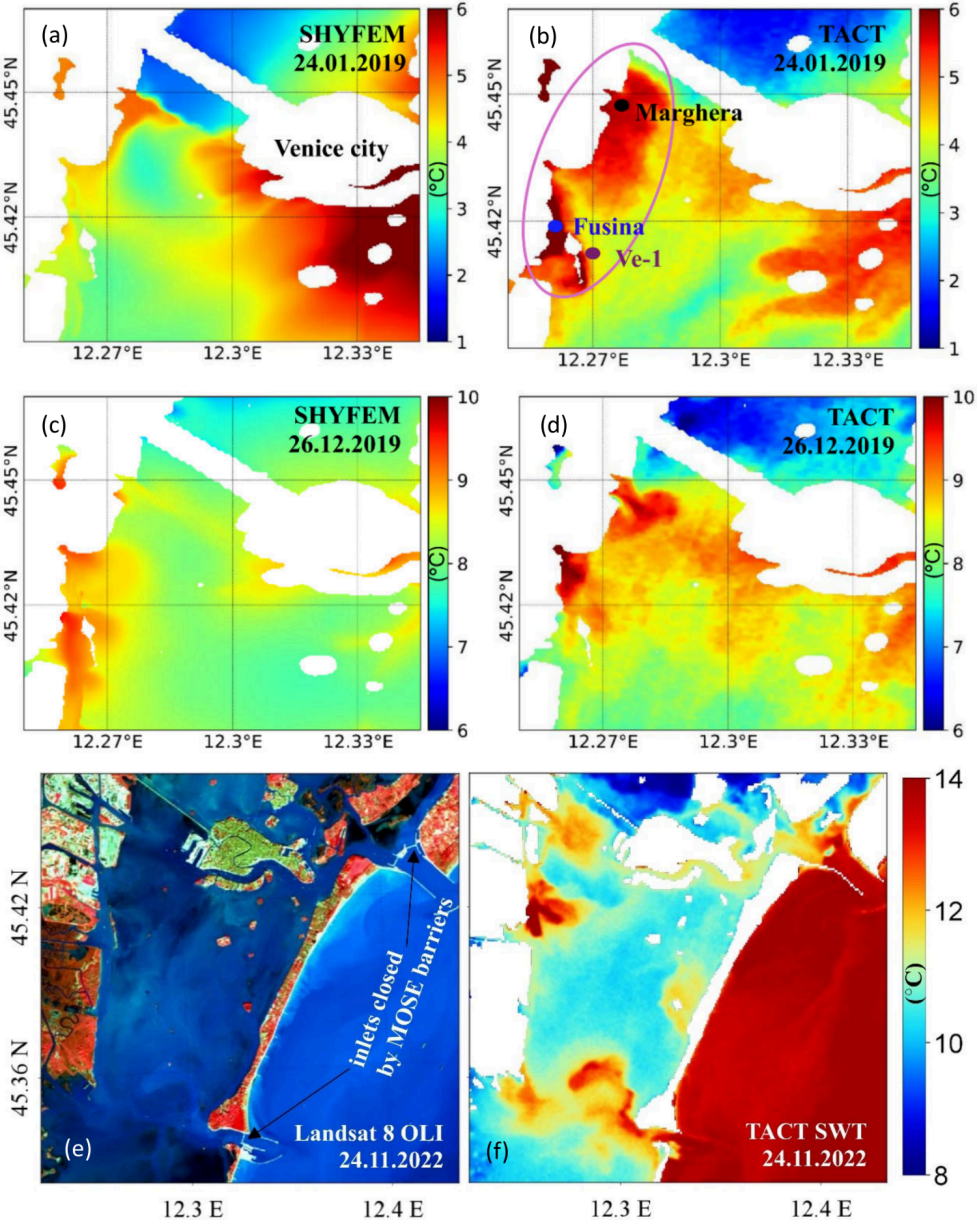
Spatial maps illustrating anthropogenic thermal influences in the Venice Lagoon. Panels (a) and (b) show SWT distributions on 24 January 2019 from SHYFEM and TACT, respectively, while panels (c) and (d) present SWT distributions on 26 December 2019 from the same products. The pink circle highlights the industrial region of Venice, including Port Marghera (black dot), the Fusina power plant outlet (blue dot), and the SAMANET station Ve-1 (brown dot). Panels (e) and (f) show the case of 24 November 2022 during MOSE barrier closure: (e) Landsat 8 OLI false color composite (865 nm – 655 nm – 560 nm) and (f) the corresponding TACT-derived SWT map.

A second case study from 24 November 2022 provided further evidence of anthropogenic influence during operation of the MOSE barriers. Figure 6e,f shows this event, when all three inlets were closed by the MOSE barriers to defend Venice from flooding. Panel (e) presents a Landsat 8 OLI composite (865 nm – 655 nm – 560 nm), and panel (f) displays the corresponding SWT map from TACT. The map shows restricted inflow of warm Adriatic water at the inlets, suggesting a thermally isolated lagoon state during the closure. In addition, a distinct thermal plume is observed at the Fusina outlet, with anomalies exceeding 6 °C above ambient temperatures, attributed to localized industrial discharge.

### 3.6 Integrated SWT Product

For integration, SHYFEM- and TACT-based SWT products were selected given their improved validation statistics (Figure 4). Further, based on the station-wise assessment, the two products were fused using a weighting approach, resulting in improved overall accuracy (see section S4 in Supporting Information). Figure 7 shows the spatial fusion of SWT fields from SHYFEM and TACT for 24 January 2019 and 26 December 2019. SHYFEM reproduced basin-scale gradients and channel–lagoon contrasts, while TACT resolved fine-scale variability including thermal plumes near the Fusina outlet and elevated temperatures around Port Marghera. The fused fields retained the local anomalies (pink circle) while embedding them within the physically consistent basin-wide gradients reproduced by SHYFEM, yielding a coherent and spatially detailed representation of winter SWT. This integration also ensured smooth transitions between anomalies and background conditions and reduced the inconsistencies that arise when relying on a single source.

**Figure 7:**
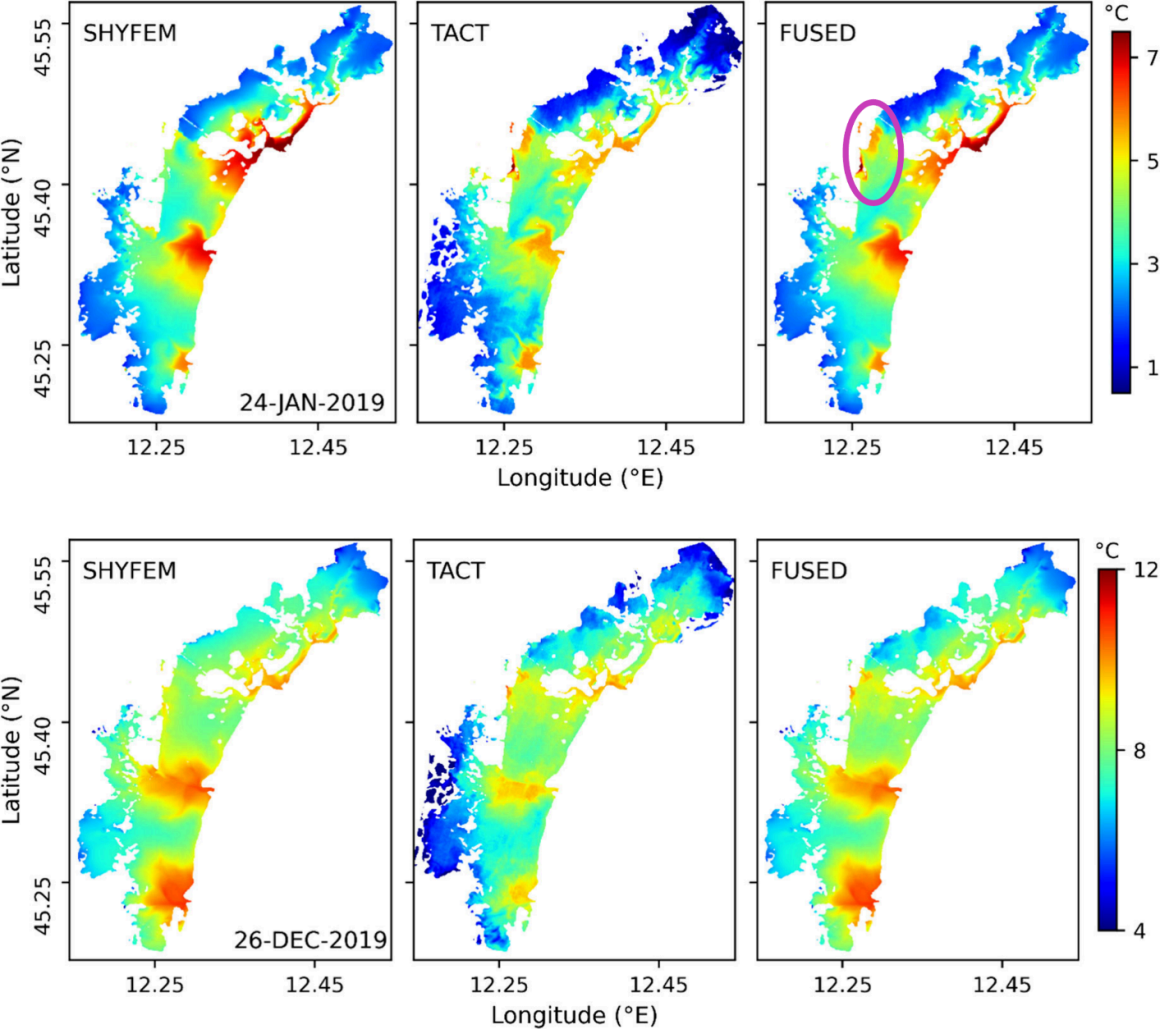
Spatial distribution of SWT from SHYFEM, TACT, and the fused product on 24 January 2019 (top) and 26 December 2019 (bottom). The pink circle highlights the industrial area where thermal anomalies are observed.

## 4 Discussion

### 4.1 Performance of SWT Products

The present study delivers a rigorous validation of multiple SWT products against dense observations from the SAMANET network and complementary ARPAV seasonal monitoring. Together these data sets provided an evaluation that spans the entire lagoon, resolving both spatial heterogeneity and seasonal dynamics, and addressing a major limitation in transitional waters where monitoring is typically sparse and uneven.^62,63^

SHYFEM showed strong agreement when assessed against SAMANET (RMSE 1.1–1.3 °C, bias approximately −0.48 °C) and ARPAV measurements (RMSE 1.52 °C, bias −0.59 °C). The higher errors were linked to localized underestimation during winter, influenced by unparameterized industrial heat discharges, uncertainties in freshwater inflows, and short-term fluctuations associated with extreme events such as the November storm surge. Despite these limitations, SHYFEM reproduced the SWT seasonal cycle with fidelity, captured subtle temporal fluctuations, and consistently represented spatial gradients across the lagoon, confirming the strength of physics based simulations when constrained by detailed real time boundary conditions and atmospheric forcing. This robustness is consistent with the recent work by,^48^ which demonstrated the capability of SHYFEM to reproduce long-term SWT time series in the Venice Lagoon, reinforcing the model’s suitability for representing thermal dynamics in transitional environments.

Among the satellite-derived products, ESA CCI showed the largest discrepancies. The limited skill of ESA CCI is in line with previous reports that its coarse resolution and gap filling methods are optimized for open ocean conditions rather than transitional waters.^16^ The data set reproduced the general intra-annual SWT cycle observed in offshore waters but lacked sensitivity to lagoonal heterogeneity and produced nearly uniform values across stations. This outcome emphasizes the importance of high spatial resolution sensors for resolving the fine scale thermal dynamics that characterize transitional environments. The Landsat-based USGS product provided this higher spatial resolution but nevertheless showed relatively large errors, consistently overestimating SWT compared to in situ observations. This overestimation is attributed to the use of MODTRAN based atmospheric parameters, including upwelling (L_u_) and downwelling (L_d_) radiances and transmittance (τ), together with the application of a lower emissivity value for water pixels. Accuracy improved substantially through the alternative thermal atmospheric correction, which employs libRadtran-derived atmospheric parameters and higher emissivity coefficients optimized for aquatic targets. The resulting TACT product, tested for the first time in the Venice Lagoon, provided the most reliable satellite estimates and improved the representation of fine-scale thermal variability. Importantly, these findings are consistent with earlier work by,^57^ where the TACT approach was validated in Belgian coastal waters and likewise demonstrated superior performance over the USGS product, supporting its robustness and transferability across aquatic environments. However, TACT performance could be further enhanced by implementing correction strategies for land adjacency effects.^30^

### 4.2 Drivers of Spatiotemporal Variability in SWT

Bathymetry is the primary driver of spatial variability in SWT in the Venice Lagoon, with shallow and deep areas exhibiting contrasting thermal responses.^64^ At shallow sites such as Ve-2 (see Figure 1b), the limited depth reduces thermal inertia and makes SWT highly sensitive to heat exchange at the air–sea interface, leading to faster cooling during winter and enhanced warming in summer. In contrast, relatively deeper channels and tidal inlets, represented by stations such as Ve-3 and Ve-8 (see Figure 1b), are strongly influenced by tidal exchange and maintain greater thermal stability, closely reflecting offshore Adriatic Sea conditions. These contrasting behaviors at shallow and deep sites have been reported in previous studies, confirming that both reduced thermal inertia and tidal renewal exert fundamental controls on SWT variability in the lagoon.^62^

This contrasting behavior was evident in the seasonal maps of SHYFEM, USGS, and TACT (Figure 5). In winter, deeper inlet channels remained relatively warmer because their greater depth and advective renewal buffered them against rapid cooling, while shallow interior flats cooled more quickly due to low thermal inertia and freshwater inflows. These differences produced horizontal gradients in SWT of up to 4–5 °C between inlet channels and the shallow interior lagoon. In summer, the situation was reversed: shallow areas warmed more strongly under intense solar radiation and limited depth, while inlet channels stayed cooler due to continuous tidal renewal with offshore waters. Spring and autumn represented transitional phases, with weaker gradients that reorganized as the system shifted toward the summer or winter state.

Taken together, these dynamics demonstrate that bathymetry and tidal exchange are the fundamental physical controls on SWT variability in the Venice Lagoon, while atmospheric forcing regulates both the intensity of horizontal gradients and their seasonal reversal between shallow flats and inlet channels.

### 4.3 Need for Multisource SWT Observations

The case studies in Figure 6a-d demonstrate how anthropogenic drivers, including industrial discharges and large-scale engineering interventions, superimpose strong localized effects on the broader thermal regime of the Venice Lagoon. The lack of explicit representation of industrial releases in SHYFEM underscores a limitation of physics-based simulations, while the ability of TACT to resolve these anomalies illustrates the complementary role of high-resolution satellite observations. A previous study by^65^ also reported thermal features near the Port Marghera region in 2013 using the Advanced Spaceborne Thermal Emission and Reflection Radiometer (ASTER). The present results confirm that these industrial plumes are a persistent feature of the lagoon. Such localized warming can modify habitat suitability and stress aquatic organisms, with potential microclimatic effects, emphasizing the importance of resolving these features for environmental monitoring and management.^66^

Satellite observations revealed the thermal signature of the MOSE closure in the lagoon. Although Figure 6e–f represents a single observation, it demonstrates the capability of TACT to provide independent reference fields that can inform hydrodynamic modeling and support the evaluation of human interventions in the lagoon system. The limited temporal sampling of Landsat 8, combined with frequent cloud cover during storm-driven events, currently prevents multievent statistical assessment. Taken together, these cases show that no single data source is sufficient, highlighting the need for combined approaches that provide continuous coverage and capture local temperature anomalies across the lagoon.

### 4.4 Integrated Framework

Integrating the SHYFEM hydrodynamic model with Landsat 8 thermal data corrected using TACT provides an effective means of capturing spatiotemporal SWT variability. The integrated SWT framework improved overall accuracy, reducing RMSE from ∼ 1.2 °C for the best individual sources (SHYFEM and TACT) to 0.99 °C for the fused field (Figure S3), thereby demonstrating the added value of integration. In addition, the fusion leveraged the strengths of two data sets already in close agreement, with spatial differences generally confined within ± 1 °C for most of the lagoon (Figure S2). As a result, the fused fields not only improved point-based validation but also enhanced the spatial and temporal coherence of SWT dynamics. By combining the large-scale physical consistency of hydrodynamic modeling with the fine-scale detail of satellite retrievals, the framework (Figure 7) captures the full range of processes governing SWT variability in the lagoon, yielding a more realistic representation of its thermal structure. Importantly, this approach establishes a basis for addressing emerging management needs, including climate adaptation planning and ecosystem assessment in transitional systems.

### 4.5 Transferability and Universal Implications

Building on openly accessible resources such as SHYFEM, Landsat, and TACT, the proposed framework is adaptable to other lagoons, estuaries, and coastal seas. SHYFEM, while optimized for the Venice Lagoon, is a flexible finite element hydrodynamic model that has been successfully applied to numerous coastal and offshore systems worldwide.^36^ In this study SHYFEM was driven by real time atmospheric and meteorological forcing, but in data-scarce regions it can be forced with global reanalysis data sets such as ERA5 or satellite derived products. Landsat 8 and 9 thermal observations provide an eight day revisit cycle at high spatial resolution, providing globally consistent thermal data that can be directly utilized in other settings. In regions lacking dense monitoring networks, cost-effective tools such as digital loggers can support SWT assessment,^67^ while open-access TACT offers additional potential for improving accuracy.

Taken together, these elements establish a transferable multi source strategy that combines sporadic in situ measurements, fine resolution satellite observations, and continuous model simulations to characterize SWT dynamics in data limited transitional environments.

### 4.6 Limitations and Future Directions

Despite the promising results, certain aspects of the framework warrant further refinement. The framework was developed and validated for the Venice Lagoon, and its application to other systems may require site-specific calibration, supported by ground-based information. In addition, data gaps from Landsat, arising from coarse revisit times and cloud cover during extreme weather conditions, restrict the ability of the fusion framework to capture event-driven thermal variability. Looking ahead, systematic testing across transitional environments with diverse settings will be essential. While the Gaussian-weighted blending strategy reduced errors and improved spatial coherence, future efforts could explore more advanced data assimilation techniques or machine-learning approaches to further optimize multisource integration. Upcoming high-resolution satellite missions such as LSTM (Land Surface Temperature Monitoring) and SatVu will help address current observational constraints and, when coupled with modeling approaches, further strengthen multisource integration. These advances will be critical for extending the framework toward operational SWT monitoring at regional to global scales.

## 5 Conclusion

Individually, in situ observations, hydrodynamic modeling, and satellite products face spatiotemporal constraints that limit their ability to fully resolve SWT dynamics in transitional environments. To address these, this study applied a comparative framework that evaluated multiple state-of-the-art SWT products, highlighting their strengths and weaknesses while identifying opportunities for improvement. The hydrodynamic model SHYFEM reproduced temporal dynamics and large-scale gradients but underestimated localized effects such as industrial discharges. The satellite-based ESA CCI product delivered gap-free daily coverage but lacked the resolution to capture lagoonal heterogeneity. In contrast, Landsat products provided fine spatial detail and successfully detected thermal plumes. Further, the alternative atmospheric correction method (TACT), tested for the first time in the Venice Lagoon, demonstrated improved accuracy relative to the standard USGS product. From the accuracy assessment, SHYFEM and TACT were identified as the best-performing sources and were integrated through a proof-of-concept fusion approach. The fused product showed reduced errors, coherent basin-scale patterns, and preserved localized anomalies, yielding a more robust representation of spatiotemporal SWT variability. Collectively, these developments demonstrate the complementary value of physics-based modeling and satellite observations and establish a transferable multisource framework for monitoring transitional waters. This framework advances our understanding of SWT dynamics and has potential applications in tracking industrial discharges, evaluating management interventions, and assessing climate-driven thermal change in coastal and estuarine systems.

## Supplementary Material



## Data Availability

Satellite data sets used in this study are publicly available, with access links provided in Table 1. Hydrodynamic model inputs and in situ observations from the SAMANET monitoring network will be made available upon request.

## Notes

Additional details on the rationale for selecting SWT products (Section S1); assessment of SHYFEM performance using the ARPAV monitoring network (Section S2), with validation results shown in Figure S1; spatial differences between SHYFEM and Landsat 8-derived SWT (Section S3); corresponding difference maps and error statistics (Figure S2) with results summarized in Table S1; and SHYFEM–TACT fusion approach and its performance evaluation (Section S4), including validation of the fused product illustrated in Figure S3 (PDF)
