# Supplementary material for: Advancing Our Understanding of Surface Water Temperature Dynamics in Transitional Environments through in Situ, Satellite, and Hydrodynamic Modeling
Source: ACS ES T Water. 2025 Nov 26;5(12):7194–206. doi: 10.1021/acsestwater.5c00583 (PMC12706785; doi:10.1021/acsestwater.5c00583)
Supplement: Supplementary file 1 [file ew5c00583_si_001.pdf]

Supporting Information

Advancing our understanding of surface water temperature dynamics in transitional environments through in-situ, satellite, and hydrodynamic modelling

Nagendra Jaiganesh Sankara Narayanan,[a*] Debora Bellafiore,[b] Francesca De Pascalis,[b] Michol Ghezzo,[b] Claire Miller,[c] Marian Scott,[c] Federica Braga,[b] Evangelos Spyrakos,[a] Andrew Tyler[a]

[a] Earth and Planetary Observation Sciences (EPOS), Biological and Environmental Sciences, Faculty of Natural Sciences, University of Stirling, Stirling, United Kingdom – FK9 4LA

[b] Institute of Marine Sciences, National Research Council, Venice, Italy – 30122

[c] School of Mathematics and Statistics, University of Glasgow, Glasgow, United Kingdom - G12 8TA

* Corresponding author - nas4@stir.ac.uk

Table of contents

Sections
S1. Rationale for the selection of SWT products
S2. Additional validation of SHYFEM
S3. Spatial differences between SHYFEM and Landsat 8 derived SWT
S4. SHYFEM+TACT fusion for improved accuracy

Figures
Figure S1. Validation of SHYFEM using the ARPAV monitoring network
Figure S2. Spatial differences between SHYFEM and Landsat 8 derived SWT and associated error maps
Figure S3. Validation of the fused product

Table
Table S1. Error statistics between SHYFEM and Landsat 8 derived SWT

S1. Rationale for the Selection of SWT Products

A wide range of hydrodynamic models are available for coastal and estuarine studies, including finite element, finite difference, and finite volume approaches.[1] For this work, we employed SHYFEM, a finite element model developed by the regional scientific community, due to its capability to resolve lagoons' complex geomorphology and hydrodynamics using a flexible unstructured grid.[2] This grid framework allows spatial discretization at variable resolutions, enabling high-fidelity representation of features such as tidal flats and deep channels,[3] while the model output can be interpolated to match the spatial resolution of diverse observational datasets for consistent intercomparison.

Among many satellite-based temperature products, the ESA Climate Change Initiative (CCI) Level 4 dataset was selected because it integrates measurements from multiple thermal and microwave satellite missions, including Sentinel-3 SLSTR (Sea and Land Surface Temperature Radiometer), to generate gap-free, daily fields.[4] While extensively validated in open-ocean contexts, its applicability to the optically and thermally heterogeneous environment of the Venice Lagoon has not been previously assessed, making it an important candidate for evaluation in this study. For clarity, all temperature datasets are referred to as surface water temperature (SWT) in this study, while original product names (e.g., ESA CCI SST) are retained only when first introduced.

To capture fine-scale thermal variability, we used Landsat 8 observations, which provide high spatial resolution and have a long record of demonstrated utility in coastal and inland water monitoring.[5] The combined operation of Landsat 8 and 9 enhances their utility by shortening the revisit time from 16 days to 8 days, minimizing temporal gaps and enabling the monitoring of meso-scale processes in coastal environments.[6] In addition to the standard USGS Level 2 product, we assessed an alternative retrieval derived using the Thermal Atmospheric Correction Tool (TACT), previously applied in Belgian coastal waters,[7] to determine whether enhanced atmospheric correction could improve retrieval accuracy under the optically complex and highly variable thermal conditions of the lagoon.

S2. Additional validation of SHYFEM

In addition to the SAMANET dataset, surface water temperature (SWT) observations from the quarterly lagoon monitoring programme coordinated by ARPAV (Regional Agency for Environmental Prevention and Protection of the Veneto) under the regional ecological status monitoring plan for the Venice Lagoon were used as an independent reference dataset for assessing SHYFEM performance. The network comprises 30 stations (violet diamonds in Figure 1a) covering diverse geomorphological and hydrodynamic settings, including shallow waters, tidal inlets, marsh edges, and areas influenced by freshwater inputs. Sampling campaigns were conducted in February (11, 12, 13, 14, 21, 25), May (14, 15, 16, 17, 23, 30), July (29, 30, 31), August (1, 9), and November (4, 5, 6, 7, 25, 26) of 2019, capturing seasonal variability in the thermal dynamics of the lagoon. Temperature was measured throughout the water column using a multiparameter profiling probe, and SWT was calculated as the average from the surface to 0.5 m depth. The dataset (will be referred as ARPAV hereafter), available from the ARPAV open data portal (https://www.arpa.veneto.it/dati-ambientali/open-data/file-e-allegati/soaml/laguna-di-venezia/dati-sonda, last accessed 28 July 2025), provides broad spatial and seasonal coverage for validation beyond the SAMANET network.

A total of 116 SWT measurements from ARPAV's quarterly lagoon monitoring were compared with SHYFEM simulations (Figure S1), covering diverse locations and seasonal conditions. SHYFEM reproduced the measured SWT with strong spatial and seasonal consistency (R^2 = 0.97, bias = −0.59 °C, RMSE = 1.52 °C, MAPE = 9.1 %), with a slight underestimation in November (purple circles). This underestimation may be linked to low atmospheric pressure recorded during the early November survey dates (average ~996 hPa), coinciding with the onset of the storm surge and subsequent flooding event of 12 November 2019, when sea levels exceeded 180 cm.[8] Such a localized extreme event can alter the lagoon circulation and heat exchange, leading to short-term temperature variations that may not be fully captured in the model results. In addition, only one Landsat 8 overpass coincided with the ARPAV sampling period (16 May 2019), yielding seven matchups between TACT-derived SWT and in situ measurements. These points (black crosses) fell along the 1:1 line, indicating excellent agreement between TACT retrievals and ARPAV observations.

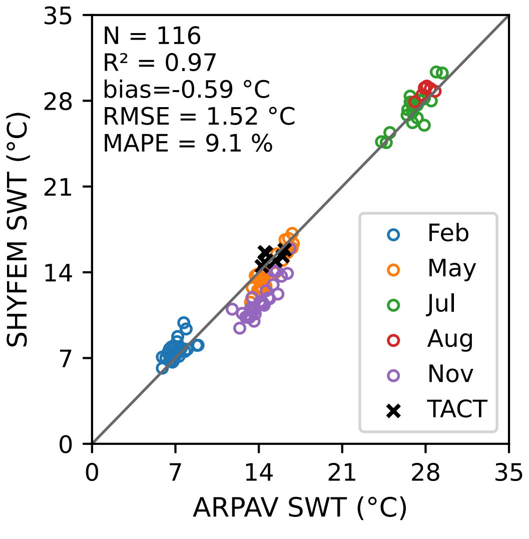

Figure S1. Validation of SHYFEM-simulated SWT against ARPAV monitoring measurements. Colours indicate months in 2019 (Feb, May, Jul, Aug, Nov) and black crosses show TACT-derived SWT for 16 May 2019. The 1:1 line is shown in grey.

S3. Spatial differences between SHYFEM and Landsat 8 derived SWT

To evaluate spatial agreement between products, pixel-wise differences and pixel-wise absolute differences were computed for each image between the Landsat 8 products and SHYFEM, as defined in Eq. (5) and Eq. (6). These were then vertically stacked, and the average of the stacks was used to derive the final spatial maps of Mean Difference (MD) and Mean Absolute Difference (MAD).

$$MD = \sum_{i=0}^{n} \frac{X_i - Z_i}{n} \quad (5)$$

$$MAD = \sum_{i=0}^{n} \left| \frac{X_i - Z_i}{n} \right| \quad (6)$$

where X_i and Z_i are the satellite-based and SHYFEM SWT values at pixel i, respectively, and n is the number of valid matched pixels.

The Mean Absolute Difference (MAD) between TACT and SHYFEM revealed minimal differences across most of the lagoon, with a spatial average of MAD equal to 0.78 °C (Table S1). However, in the region adjacent to the industrial area (highlighted by the red arrow in Figure S2 a), the difference increased significantly, reaching approximately 1–2 °C. This area is affected by thermal plumes due to cooling purposes by the production activities, and the observed discrepancy can be attributed to the fact that SHYFEM does not fully account for the discharge of warm water from the industrial area. On the other hand, the MAD map between USGS and SHYFEM (Figure S2 b) showed larger spatial differences across the lagoon, with values reaching up to 3 °C near land boundaries and a spatial average of 1.3 °C (Table S1). These discrepancies are attributed to systematic overestimations in the USGS product relative to SHYFEM. To further quantify these differences, pixel-wise Mean Differences (MD) were visualized using histograms (Figure S2 c-d). The MD between TACT and SHYFEM was confined within ± 1 °C for 94 % of valid pixels (Figure S2 c), with MD averaged over the lagoon equal to 0.13 °C and a standard deviation (SD) of 0.83 °C (Table S1). In contrast, 65 % of the USGS–SHYFEM MD values were within the range of 1–3 °C, indicating higher positive differences (Figure S2 d). The MD averaged over the lagoon was 1.14 °C, with a SD of 0.93 °C (Table S1).

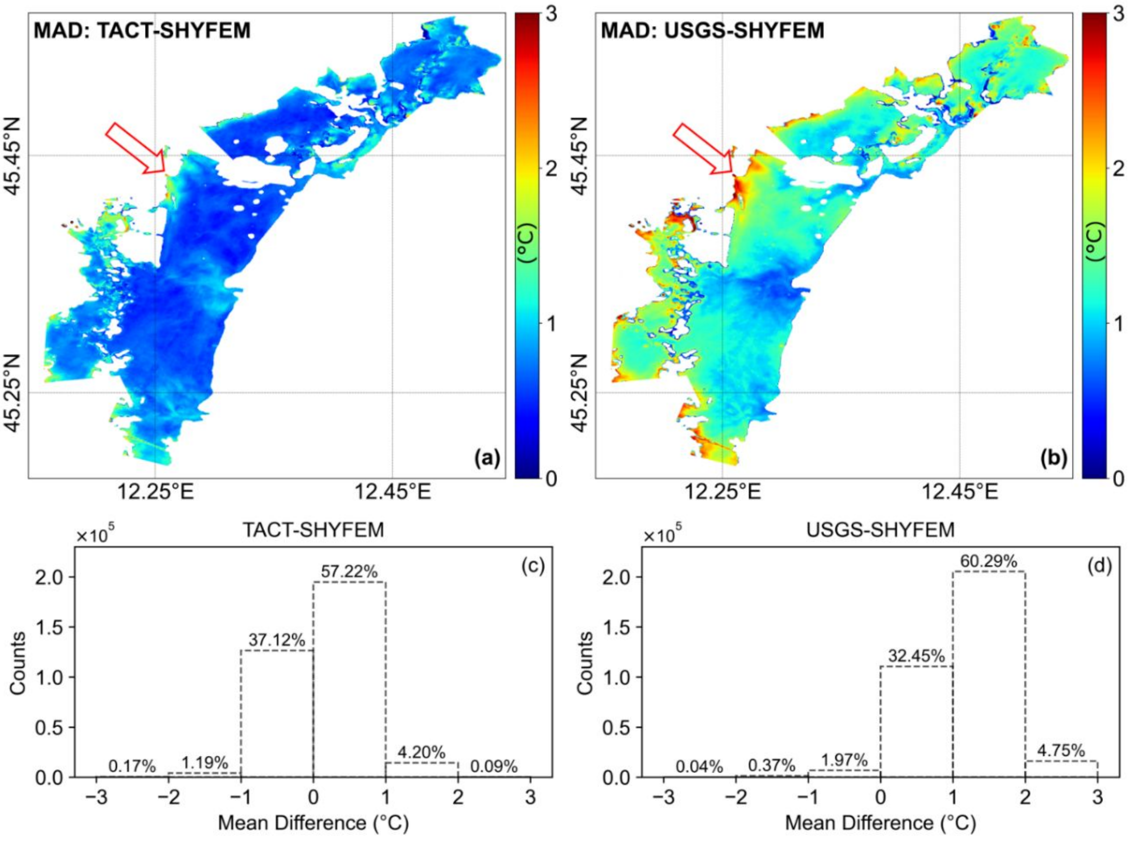

Figure S2. Spatial discrepancy maps of Landsat 8 TIRS-derived SWT compared to SHYFEM estimates. The top row shows Mean Absolute Difference (MAD), and the bottom row shows Mean Difference (MD). Panels (a) and (c) represent the comparison between TACT and SHYFEM, while panels (b) and (d) show the comparison between USGS and SHYFEM. The red arrow indicates the industrial area of Venice, including Port Marghera and a thermal power plant, which are known for their localized thermal influence on water temperature.

Table S1

Spatial differences between TACT and USGS relative to SHYFEM.

Metric	TACT-SHYFEM	USGS-SHYFEM
MAD (°C)	0.78	1.3
MD ± SD (°C)	0.13 ± 0.83	1.14 ± 0.93

S4. SHYFEM+TACT fusion for improved accuracy

For integration, SHYFEM and TACT based SWT were selected given their improved validation statistics (Figure 4), and station-wise performance was further assessed using RMSE at each site. The RMSE analysis (Figure S3 a) shows that TACT achieves the lowest errors at Ve-1 and Ve-2. Ve-1 is located near an industrial discharge zone, where TACT effectively captures localised thermal anomalies, while Ve-2 is a shallow station where satellite-derived SWT retrievals remain reliable. In contrast, SHYFEM performs better at Ve-3, Ve-7, Ve-8, and Ve-9, where SWT variability is more strongly influenced by large-scale hydrodynamic processes that are better represented by the physics-based model. Based on these results, TACT-derived SWT was used for Ve-1 and Ve-2, while SHYFEM-based SWT was applied elsewhere in the lagoon. The fused product was then compared against SAMANET observations (Figure S3 b), showing improved accuracy with a reduced RMSE of 0.99 °C, bias of −0.42 °C, and MAPE of 5.52 %.

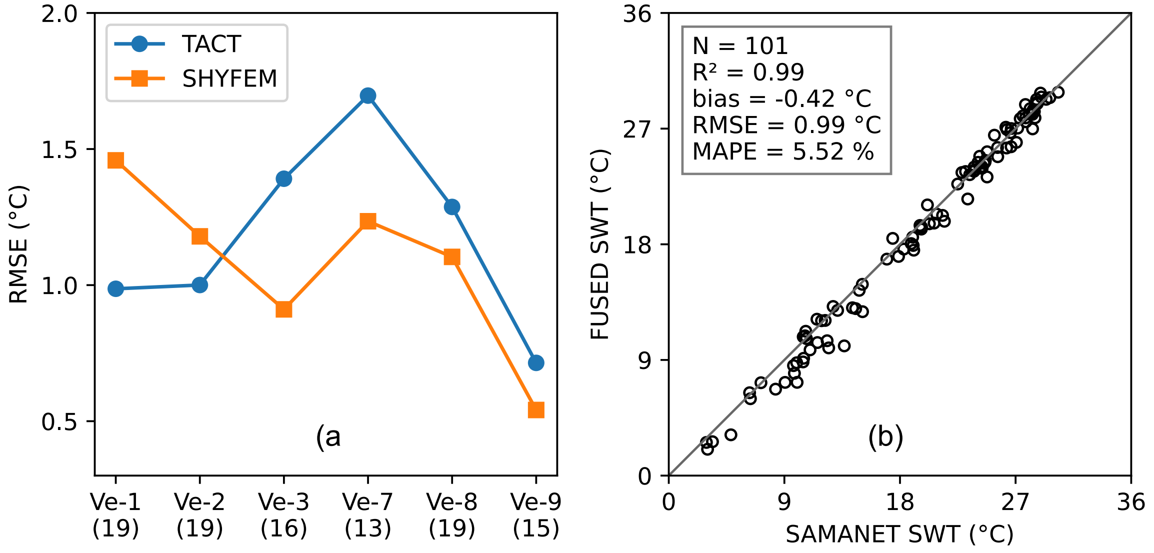

Figure S3. (a) Station-wise RMSE of TACT and SHYFEM SWT relative to SAMANET observations (numbers on the x-axis indicate the number of matchup points at each station). (b) Scatterplot of fused SWT (SHYFEM+TACT) against SAMANET observations, showing strong agreement.

References

(1) Ismail, M.; Pradhanang, S. M.; Boving, T.; Motta, S.; McCarron, B.; Volk, A. Review of Modeling Approaches at the Freshwater and Saltwater Interface in Coastal Aquifers. *Land*. Multidisciplinary Digital Publishing Institute (MDPI) August 1, 2024. https://doi.org/10.3390/land13081332.

(2) Umgiesser, G.; Ferrarin, C.; Cucco, A.; De Pascalis, F.; Bellafiore, D.; Ghezzo, M.; Bajo, M. Comparative Hydrodynamics of 10 Mediterranean Lagoons by Means of Numerical Modeling. *J. Geophys. Res. Oceans* 2014, *119* (4), 2212–2226. https://doi.org/10.1002/2013JC009512.

(3) Umgiesser, G.; Canu, D. M.; Cucco, A.; Solidoro, C. A Finite Element Model for the Venice Lagoon. Development, Set up, Calibration and Validation. *J. Mar. Syst.* 2004, *51* (1-4 SPEC. ISS.), 123–145. https://doi.org/10.1016/j.jmarsys.2004.05.009.

(4) Embury, O.; Merchant, C. J.; Good, S. A.; Rayner, N. A.; Høyer, J. L.; Atkinson, C.; Block, T.; Alerskans, E.; Pearson, K. J.; Worsfold, M.; McCarroll, N.; Donlon, C. Satellite-Based Time-Series of Sea-Surface Temperature since 1980 for Climate Applications. *Sci. Data* 2024, *11* (1). https://doi.org/10.1038/s41597-024-03147-w.

(5) Jang, J. C.; Park, K. A. High-Resolution Sea Surface Temperature Retrieval from Landsat 8 OLI/TIRS Data at Coastal Regions. *Remote Sens.* 2019, *11* (22). https://doi.org/10.3390/rs11222687.

(6) Xu, H.; Ren, M.; Lin, M. Cross-Comparison of Landsat-8 and Landsat-9 Data: A Three-Level Approach Based on Underfly Images. *GIsci. Remote Sens.* 2024, *61* (1). https://doi.org/10.1080/15481603.2024.2318071.

(7) Vanhellemont, Q.; Brewin, R. J. W.; Bresnahan, P. J.; Cyronak, T. Validation of Landsat 8 High Resolution Sea Surface Temperature Using Surfers. *Estuar. Coast. Shelf Sci.* 2022, *265*. https://doi.org/10.1016/j.ecss.2021.107650.

(8) Ferrarin, C.; Orlić, M.; Bajo, M.; Davolio, S.; Umgiesser, G.; Lionello, P. The Contribution of a Mesoscale Cyclone and Associated Meteotsunami to the Exceptional Flood in Venice on November 12, 2019. *Quarterly Journal of the Royal Meteorological Society* 2023, *149* (756), 2929–2942. https://doi.org/10.1002/qj.4539.
